# Placental Heat Shock Protein (HSP) Expression in Fetal Growth Restriction (FGR) Pregnancies: A Case–Control Immunohistochemistry Study

**DOI:** 10.3390/ijms27114841

**Published:** 2026-05-27

**Authors:** Athina A. Samara, Michel B. Janho, Konstantina Zacharouli, Theodoros Floros, Maria Ioannou, Antonios Garas, Sofia Karachrysafi, Theodora Papamitsou, Christina I. Messini, Alexandros Daponte, Sotirios Sotiriou

**Affiliations:** 1 Department of Embryology, Faculty of Medicine, University of Thessaly, Biopolis, Mezourlo, 41110 Larissa, Greece; micheljanho@live.co.uk (M.B.J.); ftheodor93@gmail.com (T.F.); sotiriousoti@yahoo.gr (S.S.); 2 Department of Pathology, Faculty of Medicine, University of Thessaly, Biopolis, Mezourlo, 41110 Larissa, Greecemioan@med.uth.gr (M.I.); 3 Department of Obstetrics and Gynecology, Faculty of Medicine, University of Thessaly, Biopolis, Mezourlo, 41110 Larissa, Greece; garasant@yahoo.gr (A.G.); messini@uth.gr (C.I.M.); daponte@uth.gr (A.D.); 4 Laboratory of Histology-Embryology, Department of Medicine, Faculty of Health Sciences, Aristotle University of Thessaloniki, 54124 Thessaloniki, Greece

**Keywords:** FGR, HSP, growth, inflammatory, IUGR

## Abstract

Fetal growth restriction (FGR) is frequently defined as the failure of the fetus to reach its genetically predetermined growth potential. Heat shock proteins (HSPs) are extreme-temperature-resistant molecules that help proteostasis. The aim of this prospective case–control immunohistochemistry study is to evaluate the expression of HSP90 and HSP70 in the placentas of pregnancies complicated with FGR and compare their levels with the control placentas of normal-growth pregnancies. A prospective case–control study was conducted including people undergoing singleton pregnancies who gave birth in a tertiary university hospital in Central Greece. Participants were divided into two equal groups: an FGR pregnancy group and a control group with normal growth. Immunohistochemistry of placental samples was assessed using anti-HSP90 alpha/beta antibody (clone F-8, Santa Cruz Biotechnology, Dallas, TX, USA) and anti-HSC70/HSP70 antibody (clone W27, sc-24, Santa Cruz Biotechnology, Dallas, TX, USA). A scoring system was created to quantify the expression of HSP90 and HSP70 in each sample, and the grade of staining was measured at four points. A total of 80 pregnant people were prospectively enrolled in our study, with 40 in each group. Both constitutive (HSP90β and HSC70/HSPA8) and stress-inducible (HSP90α and HSP70/HSPA1A/B) isoforms were analyzed. When comparing the total score of HSP expression, a statistically significant difference was observed for both HSP90 and HSP70. For HSP90 expression, only the Hofbauer cell’s stain was identified as a statistically significant independent factor, meaning that its positive expression was observed in Hofbauer cells. For HSP70 expression, only the staining of syncytiotrophoblasts was identified as an independent factor. FGR is a common pregnancy complication and a leading cause of stillbirth, neonatal mortality, and short- and long-term neonatal morbidity worldwide. Based on our findings, the lower expression levels of both HSP90 and HSP70 are associated with FGR, revealing a possible association with stress response in FGR pathophysiology. However, more robust data from larger-scale prospective studies are needed to elucidate the possible role of HSPs as potential FGR biomarkers.

## 1. Introduction

The evaluation of fetal growth represents a key factor in prenatal care. Several maternal and fetal factors can affect fetal growth, including uteroplacental function, maternal cardiovascular function and diseases, nutritional status, use of substances, perinatal infections, fetal genetic syndromes, and aneuploidy [1]. The most common cause of fetal growth restriction (FGR) is uteroplacental insufficiency that can lead to impaired growth in a normal embryo. FGR pregnancies have been linked both with an increased risk of perinatal mortality and morbidity and long-term adverse infant outcomes [2]. Prematurity-linked disorders of neurodevelopment represent a major complication of FGR; however, long-term consequences have also been described, including hypertension, diabetes mellitus, and metabolic syndrome [3].

FGR is a condition that is frequently defined as the failure of a fetus to reach its genetically predetermined growth potential. However, identifying an FGR pregnancy is not always a straightforward procedure, as fetal growth cannot be assessed through a single biometric evaluation; fetal growth is a dynamic process and its assessment requires multiple measurements over time, and growth potential is hypothetical [1,4]. Fetal size is calculated through the biometric evaluation of the head circumference, biparietal diameter, abdominal circumference (AC), and femur length and/or the derivation of estimated fetal weight (EFW) computed by different formulas [1]. FGR is divided into two categories, i.e., early < 32 and late ≥ 32 weeks of gestation, with the two main phenotypes of FGR being characterized by different clinical, ultrasound, and pathological characteristics [5]. According to the International Society of Ultrasound in Obstetrics and Gynecology (ISUOG) guidelines published in 2020 [1], the major criteria for FGR diagnosis include EFW and AC below the third centile in both instances. In early FGR pregnancies, the pulse index (PI) of umbilical artery (UA) above the 95th centile is also included. The contributory factors include AC/EFW < 10th centile, uterus artery PI >95th centile, or UA PI > 95th centile in the early category and AC/EFW < 10th centile, AC/EFW crossing centiles >two quartiles on growth centiles, cerebroplacental ratio (CPR) < 5th centile, or UA PI > 95th centile in the late category. A major contributory criterion or a combination of two contributory criteria is adequate for defining FGR. EFW alone is not sufficient to identify FGR, unless AC or EFW is below the 3rd percentile.

To date, the detection of pregnancies at risk of developing FGR is based on maternal history and ultrasound markers, and the routine screening with serum FGR biomarkers plays no role in this detection [5]. The placenta releases multiple factors into maternal circulation from the early stages of pregnancy, and the first-trimester serum levels can be associated with subsequent placenta-mediated complications [5,6]. Angiogenic factors also play a key role in the regulation of placental vascular development. Several possible biomarkers have been investigated, with placental growth factor (PlGF), a proangiogenic factor highly expressed in syncytiotrophoblasts and the maternal endothelium, being the most promising one [7]. In this context, research on novel biomarkers for the early diagnosis and prevention of FGR is crucial for decreasing the adverse events linked to FGR pregnancies.

Heat shock proteins (HSPs) are extreme-temperature-resistant molecules [8] that belong to a broader protein group in charge of proteostasis [9]. Their highly conserved nature is conveyed through their nearly unchanged presence in both eukaryotic and prokaryotic organisms over the course of time [10]. HSPs are categorized according to their molecular weight: high-molecular-weight HSPs range from 60 kDa to 100 kDa [11] and low-molecular-weight HSPs range from 20 to 27 kDa [12]. They are also classified as intracellular or extracellular HSPs [13]. Intracellular HSPs function as molecular chaperons ensuring survival in a cellular housekeeping manner [14]. These functions pertain to the alignment of newly synthesized polypeptides, repurposing of metastable proteins, assimilation of protein complexes, breakdown of misfolded proteins, and degradation of protein aggregates [8,14,15]. Extracellular HSPs, on the other hand, are expressed in response to cellular stress [16] and act as cell-to-cell mediators in the extracellular space [8]. Therefore, they play an important part in cellular homeostasis [16] as well as in the modulation of the immune system [17,18]. Furthermore, according to their molecular weight, they can trigger [19] or block [20,21] the production of proinflammatory cytokines.

The expression of HSPs can occur in various cellular locations that include the nucleus, endoplasmic reticulum, mitochondria, and cytosol [11,22,23]. In a more systematic manner, the overexpression of HSPs is correlated with poor prognosis in some cancers as they play a pivotal role in the proliferation, invasion, and death of cancer cells in tumors, thus contributing to poor prognosis [18]. The overexpression of HSPs in cancer has driven the scientific community to examine HSPs as potential disease biomarkers and tumor markers for targeted drug delivery [24]. Moreover, nanoparticles based on HSPs and designed for antitumor drug delivery were found to enhance therapeutic efficacy and reduce the adverse effects of chemotherapy albeit in cellular models [25]. However, no HSP-based drug has been developed yet for human use.

HSP70 proteins constitute a highly conserved family of molecular chaperones that are present across eukaryotic organisms [22]. In humans, the HSP70 family is encoded by approximately 17 genes, producing proteins with either stress-inducible or constitutive expression profiles. Stress-inducible HSP70s are activated in response to environmental and cellular challenges, whereas heat shock cognate HSC70 proteins are continuously expressed to support basal proteostasis [11,22,23]. These chaperone molecules contribute to the maintenance of protein homeostasis by regulating protein synthesis, folding, translocation, and degradation [24]. The functional activity of HSP70 is closely linked to its conserved domain and ATP-dependent conformational cycle. Structurally, HSP70 contains an N-terminal nucleotide-binding domain connected through a flexible linker to a C-terminal substrate-binding domain, which includes SBDα and SBDβ subdomains and terminates in an EEVD motif that mediates interactions with several cochaperones [26]. In the ADP-bound state, HSP70 binds client proteins with high affinity through the substrate-binding domain, whereas nucleotide exchange factors promote ADP release and ATP binding, leading to a lower-affinity conformation and substrate release [22,27]. Following interaction with J-domain proteins/HSP40, ATP hydrolysis restores the ADP-bound state and initiates another folding cycle, allowing substrates to undergo repeated rounds of binding and release until they achieve their native conformation [28,29].

HSP90 proteins form another highly conserved family of molecular chaperones with an approximate molecular weight of 90 kDa and are widely expressed across species, except for archaea [24,26]. In humans, six genes encode HSP90 homologues that localize to different cellular compartments. Structurally, HSP90 consists of an N-terminal nucleotide-binding domain, a middle domain involved in client protein interactions, and a C-terminal domain that mediates homodimerization and contains the MEEVD motif required for cochaperone binding [30]. Like HSP70, HSP90 functions through an ATP-dependent conformational cycle: client proteins are loaded onto the open conformation, ATP binding promotes N-terminal dimerization, ATP hydrolysis drives a more closed conformation, and ADP release returns the chaperone to its open state, enabling client maturation and release [24]. HSP90 is involved in the maturation of a substantial proportion of the human proteome, including transcription factors, ubiquitin-related proteins, and a large fraction of kinase proteins, with approximately 10% of human proteins described as HSP90 clients. Its activity is regulated by cochaperones such as AHA1 and p23, which stimulate or suppress ATPase activity, respectively, while HOP supports substrate transfer from HSP70 to HSP90 by stabilizing HSP90 in an open conformation [24,31].

Several studies indicate that multiple HSPs (HSP27, HSP40, HSP70 and HSP90) are present in placentation and decidualization processes and that their expression levels are tightly regulated during normal pregnancy [32]. An example of their important role is the fact that the glucose-regulated HSP70 isoform GRP78 (HSPA5/BiP) promotes the fusion of cytotrophoblast into syncytiotrophoblast. A balance between HSP27, HSP40 and HSP70 expression appears necessary for normal placentation and the upregulation of HSP70 and HSP90B in chorionic villi samples has been associated with placental apoptosis and increased chances of miscarriage [32]. At the same time, elevated serum or placental HSP70 levels are reported in preeclampsia and FGR and may contribute to vascular dysfunction by suppressing nitric oxide synthase [33]. In addition, HSP90 is a co-factor for endothelial nitric oxide synthase (eNOS). Placental angiogenesis requires VEGF-induced activation of eNOS via PKCδ, Akt and HSP90, and inhibitors of HSP90 diminish NO-mediated endothelial migration [34]. In the placentas of pregnancies complicated with preeclampsia, a concomitant increase in HSP70 and HSP90 along with stress-response transcription factors were observed, suggesting that the upregulation of both chaperones reflects cellular stress rather than a protective mechanism [35].

To the best of our knowledge, limited data are available regarding the role of HSPs in fetal growth and especially FGR. The aim of this prospective case–control immunohistochemistry study is to evaluate the expressions of HSP90 and HSP70 in the placentas of pregnancies complicated with FGR and compare their levels with the control placentas of normal-growth pregnancies.

## 2. Materials and Methods

### 2.1. Study Design

A prospective case–control study was conducted including people undergoing singleton pregnancies who gave birth in a tertiary university hospital in Central Greece in a two-year period. Participants were divided into two equal groups, including a group of people whose pregnancies were complicated with FGR and a control group undergoing full-term, normal-growth pregnancies. The participants in the two groups were matched based on demographics, socioeconomical status, medical history, and type of delivery. All cases included in the FGR group were diagnosed as FGR in accordance with the ISUOG guidelines for early FGR diagnosis [1]. All cases were delivered via cesarean section to match the delivery mode in the two groups. All pregnancies from the control group were full-term, while early FGR cases were diagnosed before the 32nd week of gestation, but the delivery occurred in accordance with ISUOG management guidelines using Doppler ultrasound to detect fetal distress.

The following exclusion criteria were considered: twin pregnancies, SGA pregnancies, genetic conditions of the fetus including aneuploidy, the established diagnosis of intrauterine infection, and maternal chronic diseases including hypertension, diabetes mellitus, and autoimmune diseases.

### 2.2. Data Collection

An anonymized database, including basic demographics, medical and gynecological history, and gestation information, was created.

The immunohistochemical protocol applied in this study was based on the methodology previously described by Sotiriou et al. in 2004 [36]. The placentas were obtained immediately after delivery and fixed in 10% buffered neutral formalin for at least 24 h. Afterwards, the tissues were consequently dehumidified in 50%, 80%, 90%, and 100% alcohol solutions and then in xylene solutions. Once the dehydration process was complete, the samples were placed in paraffin. Tissue sections of 4 μm thickness were sliced with the aid of a microtome, and the resulting sections underwent deparaffinization by passing through xylene and alcohol solutions followed by rinsing with distilled water. Subsequently, in vivo peroxidase activity was halted after placing the tissue sections in a 3% solution of H_2_O_2_ for 30 min. The tissue sections were washed with distilled water a second time and were submerged in tris-(hydroxymethyl) aminomethane buffered saline (tris) with a pH of 7.6. Non-specific binding to the tissues by antibodies was impeded by the incubation of tissues in 10% normal rabbit serum in tris for 30 min. This was followed by the incubation of the tissue sections from each block with primary mouse monoclonal anti-HSP90 alpha/beta antibody (clone F-8, Santa Cruz Biotechnology, Dallas, TX, USA) and mouse monoclonal anti-HSC70/HSP70 antibody (clone W27, sc-24, Santa Cruz Biotechnology, Dallas, TX, USA), in a 1:50 tris solution, for two hours. The sections were washed with tris for 5–6 min and subsequently incubated with rabbit anti-mouse peroxidase-conjugated antibody for one hour, essential for signal amplification and improved contrast. After washing for 5–6 min, the sections were further incubated with DAB plus H_2_O_2_ for five minutes, then rinsed with tris buffer and distilled water, counterstained with hematoxylin, dehydrated, and covered with Permount.

### 2.3. Outcome Measures

**Figure 1:**
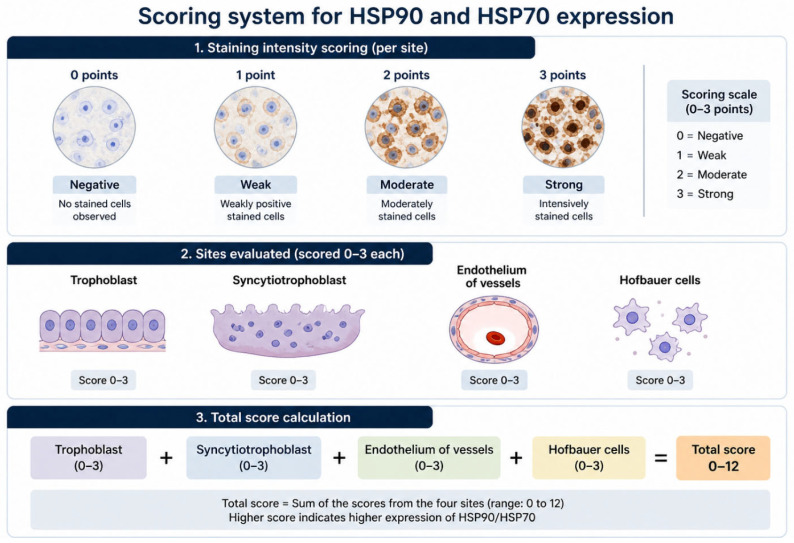
Graphical depiction of scoring system for HSP90 and HSP70 expression.

A scoring system was created to quantify the expression of HSP90 and HSP70 in each sample (Figure 1). The grade of staining was measured at four points: trophoblasts, syncytiotrophoblasts, endothelia of vessels, and Hofbauer cells. Negative or 0 points were scored when no stained cells were observed, one point for weakly positively stained cells, 2 points for moderately stained cells, and 3 points for intensely stained cells. A total score was calculated through the summation of each site’s score.

Two different investigators studied blindly and independently the samples to quantify the expression of HSP90 and HSP70 in each sample. In cases where there was a disagreement in the total score by more than one point, a third senior investigator reviewed the sample. A pilot study was conducted before to reduce interobserver variability. The four sites where the stain was measured represent the main placental elements with a biological role and were used in this study and in previous placental pathology studies.

### 2.4. Sample Calculation

An a priori power analysis was conducted for this matched case–control study including 40 cases and 40 matched controls. Assuming a two-sided α of 0.05 and a paired comparison of protein expression scores (e.g., paired *t*-test or Wilcoxon signed-rank test), this sample size provides approximately 80% power to detect a moderate within-pair effect size (standardized mean difference ≈ 0.5), assuming a within-pair correlation of approximately 0.25.

### 2.5. Statistical Analysis

Statistical analysis was carried out using R statistical environment software v. 4.4.2 (R Core Team, 2024). To compare the basic characteristics of each group, differences in the mean values between FGR and control groups were tested with the Mann–Whitney test and the χ^2^ test for categorical values. To compare the grade of expression in each site, the χ^2^ test for multiple groups was used, and to compare the mean total score between the groups, the Mann–Whitney test was performed. The Shapiro–Wilk test was performed to check for normality. The logistic regression model was used to identify factors independently associated with FGR. Multicollinearity among independent variables was assessed using the variance inflation factor (VIF) derived from a standard logistic regression model, as well as Cramér’s V for associations between categorical predictors. A VIF value > 5 was considered indicative of potentially problematic multicollinearity. A Receiver Operating Characteristic (ROC) analysis was performed to determine the sensitivity and specificity of each predictive model. The level of statistical significance was set at 0.05.

## 3. Results

A total of 80 pregnant people were prospectively enrolled in our study, with 40 in each group. The two groups were matched in terms of their basic characteristics as there were no statistically significant differences in terms of maternal age, parity, maternal BMI, smoking status, or mode of delivery.

**Figure 2:**
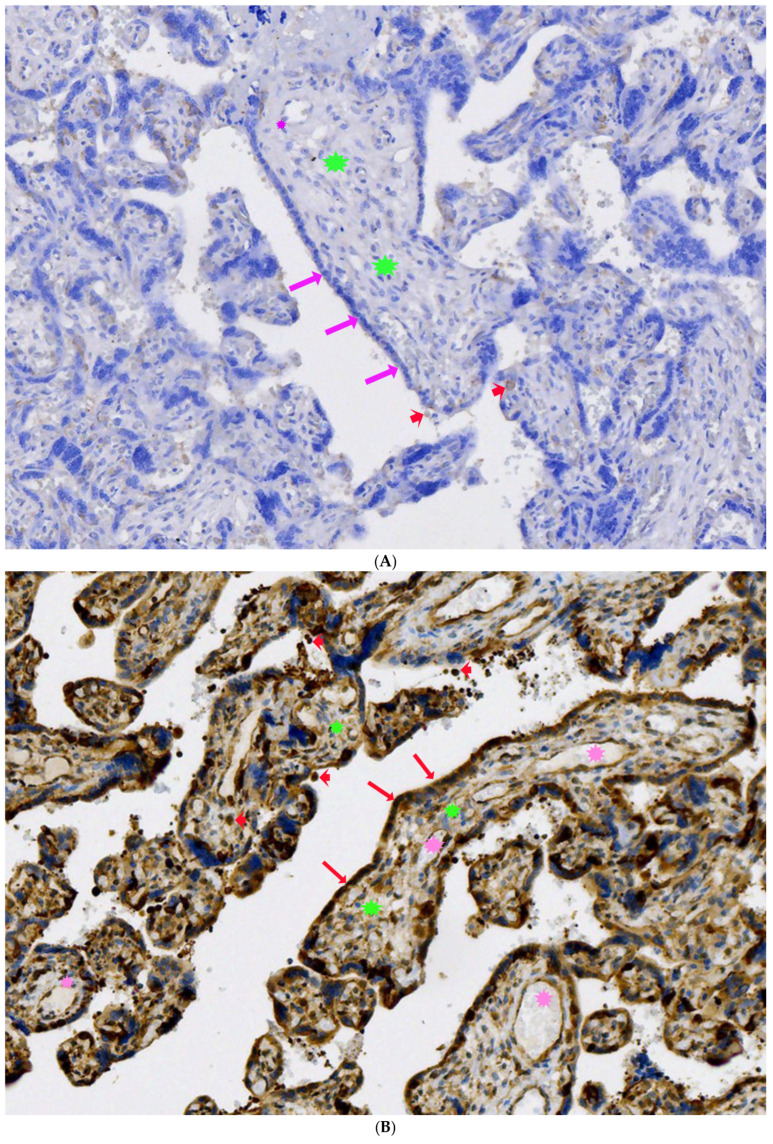
(**A**,**B**): Immunohistochemistry staining of HSP90 in (**A**) a placental sample from a pregnancy complicated with FGR and (**B**) a placental sample from a pregnancy with normal fetal growth. Purple arrows → trophoblasts, green asterisks → syncytiotrophoblasts, red arrowheads → Hofbauer cells, and purple asterisks → endothelial cells.

**Figure 3:**
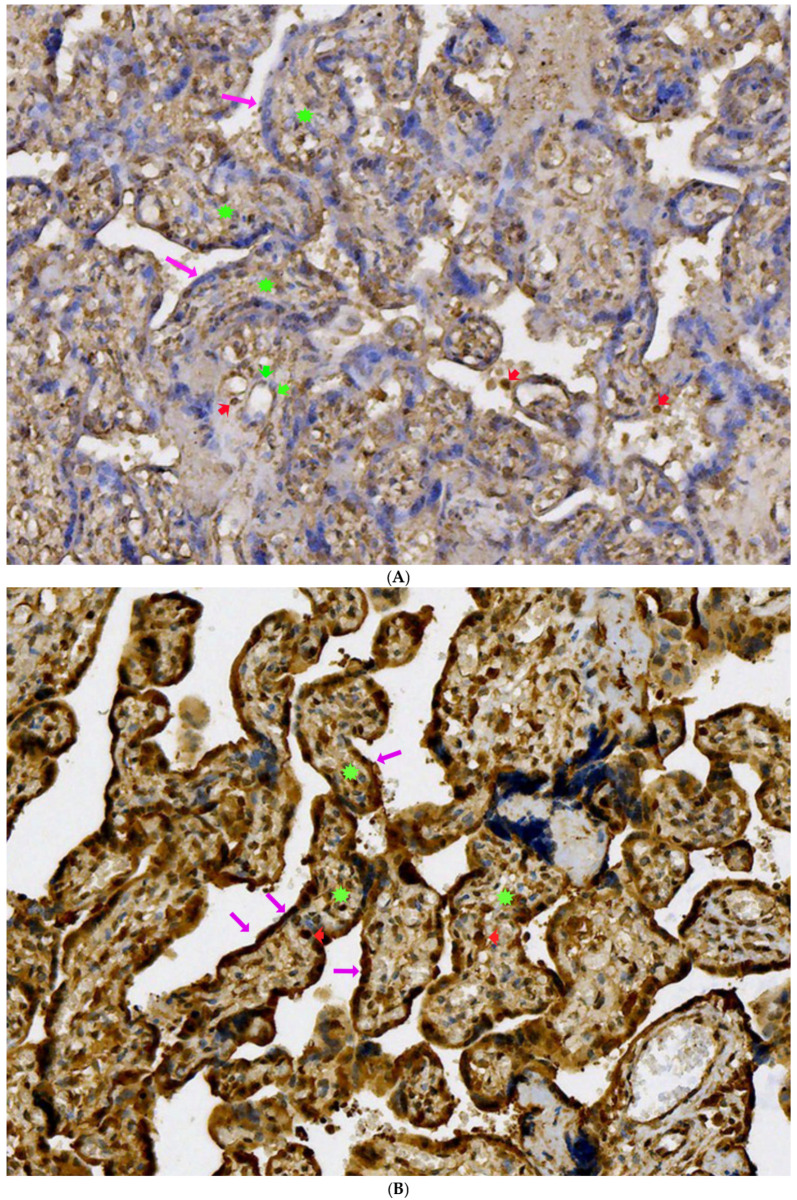
(**A**,**B**): Immunohistochemistry staining of HSP70 in (**A**) a placental sample from a pregnancy complicated with FGR and (**B**) a placental sample from a pregnancy with normal fetal growth. Purple arrows → trophoblasts, green asterisks → syncytiotrophoblasts, red arrowheads → Hofbauer cells, and green arrowheads → endothelial cells.

Both constitutive (HSP90β and HSC70/HSPA8) and stress-inducible (HSP90α and HSP70/HSPA1A/B) isoforms were analysed. When comparing the total score of HSP expression, a statistically significant difference was observed for both HSP90 and HSP70. More specifically, for HSP90, the calculated mean value was 8.00 (2.57) for the control group and 5.92 (1.51) for the FGR group (*p* = 0.001) (Figure 2A,B). In a similar manner, for HSP70 expression, the calculated mean value was 10 (0.58) for the control group and 6 (1.51) for the FGR group (*p*< 0.001) (Figure 3A,B).

Multicollinearity was observed between trophoblasts and syncytiotrophoblasts. The VIF values for these variables were 5.10 and 5.41, respectively, exceeding the predefined threshold. Additionally, Cramér’s V between trophoblasts and syncytiotrophoblasts was 0.79, indicating a very strong association. Other variables showed lower VIF values suggesting less pronounced collinearity. Due to strong collinearity, only syncytiotrophoblasts were retained in the final multivariable model based on their stronger univariable association and biological relevance.

To identify independent predictive factors associated with FGR, a regression model was applied. For HSP90 expression, only the Hofbauer cell’s stain was identified as a statistically significant independent factor (OR = 0.13, *p* = 0.001), meaning that positive expression in Hofbauer cells is 87% more possible in normal-growth pregnancies when compared with FGR pregnancies. For HSP70 expression, only the stain in syncytiotrophoblasts was identified as an independent factor (OR = 0.61, *p* = 0.04), meaning that positive expression in trophoblast cells is 39% more possible in normal-growth pregnancies when compared with FGR pregnancies.

**Figure 4:**
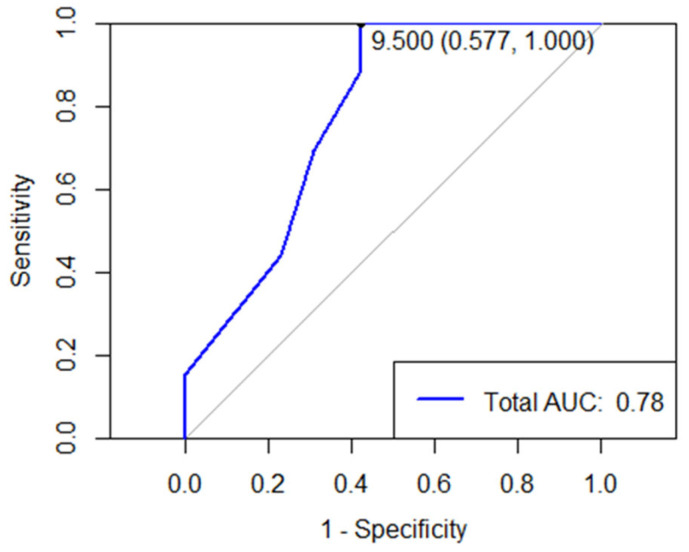
ROC curve for total score of HSP90.

Figure 4 represents the ROC curve for the total score of HSP90 expression. AUC was calculated to be 0.78, and the optimal threshold was 9.5 (with 58% sensitivity and 100% specificity). These results can be interpreted as each point of increase in the total score reduces the chance of FGR by 44% (OR: 0.56, CI: 0.41, 0.72); alternatively (1/0.56 = 1.79), each point of reduction in the total score increases the chance of FGR by 79% (OR: 1.79, CI: 1.4, 2.4).

**Figure 5:**
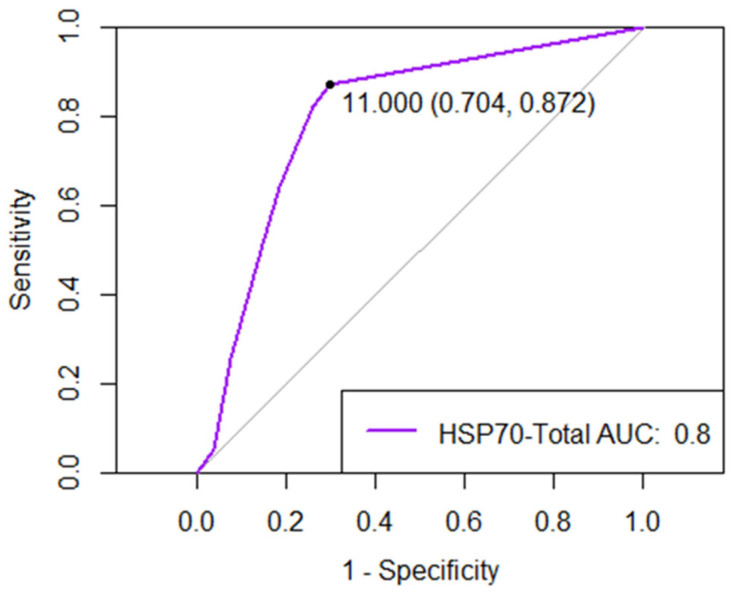
ROC curve for total score of HSP70.

Figure 5 depicts the ROC curve for HSP70 expression. AUC was measured to be 0.8, and a cutoff value of 11 is associated with 70% specificity and 87% sensitivity. Similar to HSP90, each point of increase in the HSP70 total score reduces the chance of IUGR by 40% (OR: 0.6, CI: 0.45, −0.76); alternatively, each point of reduction in the HSP70 total score results in 67% increased possibility of FGR occurrence (OR: 1.67, CI: 1.3, −2.2).

## 4. Discussion

This study investigated the possible role of HSP90 and HSP70 in the pathophysiology of FGR, by examining the expression of these proteins in placental samples. Based on our findings, HSPs appear to contribute to the maintenance of placental cellular homeostasis through their chaperone activity. Furthermore, the placentas from pregnancies with fetal growth restriction demonstrated significantly increased expression of both HSP90 and HSP70 when compared with the placentas from normal-growth pregnancies. The total expression of both HSPs was statistically significantly higher in normal-growth pregnancies when compared with FGR pregnancies. More specifically, in logistic regression, HSP90 expression of Hofbauer cells was found to be independently associated with fetal growth, and its higher expression was linked with normal growth. Regarding HSP70, higher expression in syncytiotrophoblasts was independently associated with normal growth, and low levels of its expression were statistically significantly found in pregnancies complicated with FGR.

Hofbauer cells (HBCs) are placental macrophages with a spherical, fusiform, or star-like appearance and are found in a plethora of pleomorphic cells in the villous stroma. The size of their processes determines their length, and their diameter can range from 10 to 30 μm [37]. HBCs contribute majorly to placental morphogenesis as well as homeostasis. In the first trimester, it is believed that a subset of HBCs is involved in cellular debris removal during the early stages of placental development [38]. Furthermore, they orchestrate the functions of endothelia and trophoblasts through paracrine signaling and cell-to-cell communication [39,40]. An in vitro study by Khan et al. examining the role of macrophages in trophoblast growth reported that cultures of trophoblast cells treated with HBC supernatant resulted in enhanced trophoblast proliferation and growth in comparison to the same cultures treated with macrophages from the peritoneal cavity [39]. This indicates that HBCs produce a plethora of cytokines that can contribute to the growth of placental tissue. Additionally, their pro-angiogenesis role is mediated via their high expression of vascular endothelial growth factor including its receptors (VEGF) [40], fibroblast growth factor 2 (FGF-2), and osteopontin (OPN) [41] that promote placental growth and angiogenesis. HBCs also produce Sprout proteins, which, in turn, contribute to the vascularization and the differentiation of placental villi [42].

Towards the second and eventually the third trimester of pregnancy, HBCs mature into a subset responsible for the downregulation of inflammation and immunosurveillance [43]. Approximately one-third of the HBC population near the end of the third trimester belongs to a subset of HBCs that is characterized by the secretion of IL6 and TNFa stimulation by immune complexes [44]. Also, term HBCs respond, in a cytotoxic manner, to proinflammatory incidents or pathogens originating from the maternal side by secreting nitric oxide [45]. Understanding the protective role of HBCs against inflammation and their action as promoters of vascularization is crucial to explain the significance of HSP90 expression in HBCs. HSP90 as chaperones are highly expressed in HBCs to play a protective role in HBCs and placental macrophages and to preserve cell homeostasis.

Placentation is the formation as well as the proper functioning of the placenta. The placenta plays a vital role in several complex and vital fetal–maternal functions and is crucial in the maintenance of pregnancy and the health of both the developing fetus and its mother. Poor placentation can lead to unfavorable pregnancy outcomes such as preeclampsia and FGR [32]. Environmental and pathological stressors, including hypoxia/ischemia, inflammation, oxidative stress, tissue injury, and genetic factors, induce protein misfolding and activate the unfolded protein response (UPR), resulting in ER stress and the disruption of normal protein folding and processing [46,47]. Persistent ER stress promotes protein aggregation, cytotoxicity, inflammatory activation, and oxidative stress, which collectively impair spiral artery remodeling and placental perfusion [48]. The figure compares normal pregnancy, characterized by adequate maternal–fetal blood flow and proper villous perfusion, with preeclampsia/fetal growth restriction (FGR), where impaired placental circulation is associated with hypoxia, increased reactive oxygen species (ROS) production, and inflammatory signaling [49,50,51,52].

The current literature lacks a consensus regarding the accurate biomarkers with their diagnostic and prognostic values for placental ischemic disease, and its diagnosis is mainly based on ultrasonographical features [52]. In recent decades, many researchers have investigated the potential role of various molecules associated with cellular stress in various histopathological changes in the placentas of FGR fetuses [52]. A few studies have explored the utility of HSPs in this niche setting. A case–control study in 2006 that recruited 149 pregnant women with hypertensive disorders found that their HSP70 levels were elevated in contrast to those of the control group [53]. Noteworthily, no difference in HSP70 serum levels was noted in the group of women with preeclampsia at all stages of preeclampsia [54].

Over the years, several studies have investigated the role of HSPs in placental pathology and their relationship with inflammatory processes [52]. Placental inflammation contributes to endothelial dysfunction, which is an important part of placental insufficiency. The association between HSP70 and proinflammatory cytokines of monocytes exposed to Toll-like receptor (TLR) agonists suggests that HSP70 can negatively regulate inflammatory responses of the placenta [55]. Additionally, high HSP70 serum levels in treatment-resistant preterm delivery pregnancies have been reported in some cases, indicating that HSP70 may be a useful marker for evaluating the curative effects of treatment for preterm delivery [56]. These findings underline the key role of HSPs in negative feedback with inflammatory pathways activated in the placentas in stress-induced situations [52].

A large amount of conflicting research exists in the literature that explores the associations of HSPs with preeclampsia-complicated pregnancies. While extracellular HSP60 and HSP70 were found to be positively correlated with hepatic dysfunction markers such as glutamic pyruvic transaminase (GPT), glutamic oxaloacetic transaminase (GOT), uric acid, and lactic dehydrogenase (LDH), as well as inflammatory markers such as IL-1β and TNFα, in people with preeclampsia-complicated pregnancies [57], other studies argue that this correlation is not indicative of intracellular HSP levels; therefore, such associations should be interpreted with caution [58]. Thus, other studies aimed to elicit the role of HSP’s with various changes in the placentas of FGR fetuses as verified by histopathology. Higher expression of HSP70 and endothelial NO synthase (eNOS) was found in 135 specimens of placental villi of preeclamptic and FGR pregnancies in comparison to healthy controls using an immunostaining assay [59]. Further histopathological findings in another study of FGR placentas report that the presence of excessive syncytial knots, villi with no vascularization, or thrombi expressed more HSPs, of all subtypes, in comparison to healthy controls [59]. Noteworthily, zones that present infarction expressed lower levels of HSPs than other zones in the placentas [59].

The induction of preterm delivery is also thought to be mediated by HSPs. In a case series of 12 preterm deliveries, anti-HSP antibodies (of both HSP60 and HSP70) were detected, and the authors suggest that these antibodies are a key contributor to preterm delivery [60]. On the other hand, conflicting results were reported in a study of 19 pregnancies [61] (5 healthy, 5 hypertensive, 5 IUGR, and 4 comorbid hypertensive and IUGR pregnancies) as no difference in HSP70 expression was found. The authors further argue that the use of HSP70 as a biomarker of oxidative stress should be avoided [62].

To date, there are no generally accepted biomarkers that can be used for the screening and monitoring of FGR pregnancies. Biomarkers such as HSPs could be useful especially in cases where the current sonographic criteria fail to correctly categorize pregnancies at risk of complications due to placental dysfunction. The integration of new knowledge is critical to the development of algorithms that can individualize the approach of a patient based on their particular needs. Moreover, the ROC analysis and threshold values were used to better evaluate the association between FGR and HSP90 and HSP70. As placental samples are difficult to obtain before delivery, the HSP serum levels can be used for diagnostic, screening, and monitoring purposes. The published evidence has already associated HSP serum levels with placental insufficiency-linked conditions such as preeclampsia [57]. Biomarkers such as HSP serum levels may be useful especially in cases where the current sonographic diagnostic criteria for FGR diagnosis and management fail to correctly categorize pregnancies at risk of complications [63]. However, there is still a long way to go until a more robust conclusion can be drawn regarding their role in clinical practice. One important feature is the fact that most of the studies examining the role of HSPs have examined limited samples. Therefore, larger-scale studies are needed so that we can draw conclusions with an acceptable level of statistical significance. Moreover, the different methodological protocols used by different research groups can also be a source of confusion. Thus, a commonly accepted methodology needs to be applied.

Before the generalization of our findings, several strengths and limitations of our study must be considered. The strengths of this study include the strict criteria for FGR diagnosis based on the international consensus applied, the blind case–control design for the evaluation of immunohistochemistry results, and the low heterogeneity. On the other hand, the small sample size remains a drawback for the generalization of our findings, increasing the risk of statistical model overfitting. However, collinearity tests were used, and associations between values were considered. Moreover, FGR represents a heterogeneous condition with distinct pathophysiological mechanisms, and the absence of detailed subgroup stratification, including early- versus late-onset FGR and Doppler characteristics, may limit the interpretation of the findings.

## 5. Conclusions

FGR is a common pregnancy complication and a leading cause of stillbirth, neonatal mortality, and short- and long-term neonatal morbidity worldwide. Based on our findings, the lower expression levels of both HSP90 and HSP70 are associated with FGR, revealing a possible association with stress response in FGR pathophysiology. However, more robust data from larger-scale prospective studies are needed to elucidate the possible role of HSPs as potential FGR biomarkers.

## Data Availability

The data presented in this study are available on request from the corresponding author due to patient privacy considerations.

## References

[1] Lees C.C., Stampalija T., Baschat A., da Silva Costa F., Ferrazzi E., Figueras F., Hecher K., Kingdom J., Poon L.C., Salomon L.J. (2020). ISUOG Practice Guidelines: Diagnosis and management of small-for-gestational-age fetus and fetal growth restriction. Ultrasound Obstet. Gynecol..

[2] Miller S.L., Huppi P.S., Mallard C. (2016). The consequences of fetal growth restriction on brain structure and neurodevelopmental outcome. J. Physiol..

[3] Nohuz E., Rivière O., Coste K., Vendittelli F. (2020). Prenatal identification of small-for-gestational age and risk of neonatal morbidity and stillbirth. Ultrasound Obstet. Gynecol..

[4] Samara A.A., Floros T., Zacharouli K., Ioannou M., Stamouli D., Janho M., Skentou C., Daponte A., Sotiriou S. (2025). Morphological Macroscopical Placental Features of Pregnancies Complicated with Fetal Growth Restriction: A Case-Control Study. Maedica.

[5] Melamed N., Baschat A., Yinon Y., Athanasiadis A., Mecacci F., Figueras F., Berghella V., Nazareth A., Tahlak M., McIntyre H.D. (2021). FIGO (international Federation of Gynecology and obstetrics) initiative on fetal growth: Best practice advice for screening, diagnosis, and management of fetal growth restriction. Int. J. Gynaecol. Obstet..

[6] Smith G.C. (2004). First trimester origins of fetal growth impairment. Semin. Perinatol..

[7] Wang A., Rana S., Karumanchi S.A. (2009). Preeclampsia: The role of angiogenic factors in its pathogenesis. Physiology.

[8] De Maio A. (2011). Extracellular heat shock proteins, cellular export vesicles, and the stress observation system: A form of communication during injury, infection, and cell damage. It is never known how far a controversial finding will go! Dedicated to Ferruccio Ritossa. Cell Stress. Chaperones.

[9] Hartl F.U., Bracher A., Hayer-Hartl M. (2011). Molecular chaperones in protein folding and proteostasis. Nature.

[10] Figaj D. (2025). The Role of Heat Shock Protein (Hsp) Chaperones in Environmental Stress Adaptation and Virulence of Plant Pathogenic Bacteria. Int. J. Mol. Sci..

[11] Daugaard M., Rohde M., Jaattela M. (2007). The heat shock protein 70 family: Highly homologous proteins with overlapping and distinct functions. FEBS Lett..

[12] Gusev N.B., Bukach O.V., Marston S.B. (2005). Structure, properties, and probable physiological role of small heat shock protein with molecular mass 20 kD (Hsp20, HspB6). Biochemistry.

[13] Schmitt E., Gehrmann M., Brunet M., Multhoff G., Garrido C. (2007). Intracellular and extracellular functions of heat shock proteins: Repercussions in cancer therapy. J. Leukoc. Biol..

[14] Saibil H. (2013). Chaperone machines for protein folding, unfolding and disaggregation. Nat. Rev. Mol. Cell Biol..

[15] Verghese J., Abrams J., Wang Y., Morano K.A. (2012). Biology of the heat shock response and protein chaperones: Budding yeast (*Saccharomyces cerevisiae*) as a model system. Microbiol. Mol. Biol. Rev. MMBR.

[16] Rylander M.N., Feng Y., Bass J., Diller K.R. (2005). Thermally induced injury and heat-shock protein expression in cells and tissues. Ann. N. Y. Acad. Sci..

[17] Wallin R.P., Lundqvist A., More S.H., von Bonin A., Kiessling R., Ljunggren H.G. (2002). Heat-shock proteins as activators of the innate immune system. Trends Immunol..

[18] Zininga T., Ramatsui L., Shonhai A. (2018). Heat shock proteins as immunomodulants. Molecules.

[19] Ahmed K.A., Xiang J. (2011). Mechanisms of cellular communication through intercellular protein transfer. J. Cell Mol. Med..

[20] Beere H.M. (2004). “The stress of dying”: The role of heat shock proteins in the regulation of apoptosis. J. Cell Sci..

[21] Hadadi E., Zhang B., Baidzajevas K., Yusof N., Puan K.J., Ong S.M., Yeap W.H., Rotzschke O., Kiss-Toth E., Wilson H. (2016). Differential IL-1beta secretion by monocyte subsets is regulated by Hsp27 through modulating mRNA stability. Sci. Rep..

[22] Rosenzweig R., Nillegoda N.B., Mayer M.P., Bukau B. (2019). The Hsp70 chaperone network. Nat. Rev. Mol. Cell Biol..

[23] Brocchieri L., Conway de Macario E., Macario A.J. (2008). hsp70 genes in the human genome: Conservation and differentiation patterns predict a wide array of overlapping and specialized functions. BMC Evol. Biol..

[24] Hu C., Yang J., Qi Z., Wu H., Wang B., Zou F., Mei H., Liu J., Wang W., Liu Q. (2022). Heat shock proteins: Biological functions, pathological roles, and therapeutic opportunities. MedComm.

[25] Shi K., Wang Y., Zhou X., Gui H., Xu N., Wu S., He C., Zhao Z. (2020). Tumor microenvironment targeting with dual stimuli-responsive nanoparticles based on small heat shock proteins for antitumor drug delivery. Acta Biomater..

[26] Johnson J.L. (2012). Evolution and function of diverse Hsp90 homologs and cochaperone proteins. Biochim. Biophys. Acta.

[27] Brehmer D., Rüdiger S., Gässler C.S., Klostermeier D., Packschies L., Reinstein J., Mayer M.P., Bukau B. (2001). Tuning of chaperone activity of Hsp70 proteins by modulation of nucleotide exchange. Nat. Struct. Biol..

[28] Kityk R., Kopp J., Mayer M.P. (2018). Molecular mechanism of J-domain-triggered ATP hydrolysis by Hsp70 Chaperones. Mol. Cell..

[29] Kityk R., Kopp J., Sinning I., Mayer M.P. (2012). Structure and dynamics of the ATP-bound open conformation of Hsp70 chaperones. Mol. Cell..

[30] Ali M.M., Roe S.M., Vaughan C.K., Meyer P., Panaretou B., Piper P.W., Prodromou C., Pearl L.H. (2006). Crystal structure of an Hsp90-nucleotide-p23/Sba1 closed chaperone complex. Nature.

[31] Zierer B.K., Rubbelke M., Tippel F., Madl T., Schopf F.H., Rutz D.A., Richter K., Sattler M., Buchner J. (2016). Importance of cycle timing for the function of the molecular chaperone Hsp90. Nat. Struct. Mol. Biol..

[32] Jee B., Dhar R., Singh S., Karmakar S. (2021). Heat Shock Proteins and Their Role in Pregnancy: Redefining the Function of “Old Rum in a New Bottle”. Front. Cell Dev. Biol..

[33] Lai H., Nie L., Zeng X., Xin S., Wu M., Yang B., Luo Y., Liu B., Zheng J., Liu H. (2022). Enhancement of heat shock protein 70 attenuates inducible nitric oxide synthase in preeclampsia complicated with fetal growth restriction. J. Matern. Fetal Neonatal Med..

[34] Krause B.J., Hanson M.A., Casanello P. (2011). Role of nitric oxide in placental vascular development and function. Placenta.

[35] Padmini E., Venkatraman U., Srinivasan L. (2012). Mechanism of JNK signal regulation by placental HSP70 and HSP90 in endothelial cell during preeclampsia. Toxicol. Mech. Methods..

[36] Sotiriou S., Liatsos K., Ladopoulos I., Arvanitis D.L. (2004). A comparison in concentration of heat shock proteins (HSP) 70 and 90 on chorionic villi of human placenta in normal pregnancies and in missed miscarriages. Clin. Exp. Obstet. Gynecol..

[37] King B.F. (1987). Ultrastructural differentiation of stromal and vascular components in early macaque placental villi. Am. J. Anat..

[38] Wood G.W., King C.R. (1982). Trapping antigen-antibody complexes within the human placenta. Cell Immunol..

[39] Khan S., Katabuchi H., Araki M., Nishimura R., Okamura H. (2000). Human Villous Macrophage-Conditioned Media Enhance Human Trophoblast Growth and Differentiation In Vitro. Biol. Reprod..

[40] Demir R., Kayisli U.A., Seval Y., Celik-Ozenci C., Korgun E.T., Demir-Weusten A.Y., Huppertz B. (2004). Sequential Expression of VEGF and its Receptors in Human Placental Villi During Very Early Pregnancy: Differences Between Placental Vasculogenesis and Angiogenesis. Placenta.

[41] Johnson G.A., Burghardt R.C., Bazer F.W., Spencer T.E. (2003). Osteopontin: Roles in Implantation and Placentation. Biol. Reprod..

[42] Anteby E.Y., Natanson-Yaron S., Greenfield C., Goldman-Wohl D., Haimov-Kochman R., Holzer H., Yagel S. (2005). Human Placental Hofbauer Cells Express Sprouty Proteins: A Possible Modulating Mechanism of Villous Branching. Placenta.

[43] Bezemer R.E., Faas M.M., Van Goor H., Gordijn S.J., Prins J.R. (2024). Decidual macrophages and Hofbauer cells in fetal growth restriction. Front. Immunol..

[44] Swieboda D., Johnson E.L., Beaver J., Haddad L., Enninga E.A.L., Hathcock M., Cordes S., Jean V., Lane I., Skountzou I. (2020). Baby’s First Macrophage: Temporal Regulation of Hofbauer Cell Phenotype Influences Ligand-Mediated Innate Immune Responses across Gestation. J. Immunol..

[45] Myatt L., Eis A.L.W., Brockman D.E., Kossenjans W., Greer I., Lyall F. (1997). Inducible (type II) nitric oxide synthase in human placental villous tissue of normotensive, pre-eclamptic and intrauterine growth-restricted pregnancies. Placenta.

[46] Yung H.W., Atkinson D., Campion-Smith T., Olovsson M., Charnock-Jones D.S., Burton G.J. (2014). Differential activation of placental unfolded protein response pathways implies heterogeneity in causation of early- and late-onset pre-eclampsia. J. Pathol..

[47] Bastida-Ruiz D., Aguilar E., Ditisheim A., Yart L., Cohen M. (2017). Endoplasmic reticulum stress responses in placentation—A true balancing act. Placenta.

[48] Guzel E., Arlier S., Guzeloglu-Kayisli O., Tabak M.S., Ekiz T., Semerci N., Larsen K., Schatz F., Lockwood C.J., Kayisli U.A. (2017). Endoplasmic Reticulum Stress and Homeostasis in Reproductive Physiology and Pathology. Int. J. Mol. Sci..

[49] Singh M.K., Han S., Ju S., Ranbhise J.S., Ha J., Yeo S.G., Kim S.S., Kang I. (2025). Hsp70: A Multifunctional Chaperone in Maintaining Proteostasis and Its Implications in Human Disease. Cells.

[50] Vornic I., Buciu V., Furau C.G., Gaje P.N., Ceausu R.A., Dumitru C.S., Barb A.C., Novacescu D., Cumpanas A.A., Latcu S.C. (2024). Oxidative Stress and Placental Pathogenesis: A Contemporary Overview of Potential Biomarkers and Emerging Therapeutics. Int. J. Mol. Sci..

[51] Taipale M., Jarosz D.F., Lindquist S. (2010). HSP90 at the Hub of Protein Homeostasis: Emerging Mechanistic Insights. Nat. Rev. Mol. Cell Biol..

[52] Samara A.A., Lafioniatis A., Ioannou M., Tsiapakidou S., Gerede A., Anastasakis E., Daponte A., Sotiriou S. (2025). The role of heat shock proteins in placental ischemic disease: A narrative review of the current literature. Int. J. Gynaecol. Obstet..

[53] Molvarec A., Prohászka Z., Nagy B., Szalay J., Füst G., Karádi I., Rigó J. (2006). Association of elevated serum heat-shock protein 70 concentration with transient hypertension of pregnancy, preeclampsia and superimposed preeclampsia: A case-control study. J. Hum. Hypertens..

[54] Molvarec A., Prohászka Z., Nagy B., Kalabay L., Szalay J., Füst G., Karádi I., Rigó J. (2007). Association of increased serum heat shock protein 70 and C-reactive protein concentrations and decreased serum alpha(2)-HS glycoprotein concentration with the syndrome of hemolysis, elevated liver enzymes, and low platelet count. J. Reprod. Immunol..

[55] Ferat-Osorio E., Sánchez-Anaya A., Gutiérrez-Mendoza M., Boscó-Gárate I., Wong-Baeza I., Pastelin-Palacios R., Pedraza-Alva G., Bonifaz L.C., Cortés-Reynosa P., Pérez-Salazar E. (2014). Heat shock protein 70 down-regulates the production of toll-like receptor-induced pro-inflammatory cytokines by a heat shock factor-1/constitutive heat shock element-binding factor-dependent mechanism. J. Inflamm..

[56] Fukushima A., Kawahara H., Isurugi C., Syoji T., Oyama R., Sugiyama T., Horiuchi S. (2005). Changes in serum levels of heat shock protein 70 in preterm delivery and pre-eclampsia. J. Obstet. Gynaecol. Res..

[57] Álvarez-Cabrera M.C., Barrientos-Galeana E., Barrera-García A., Osorio-Caballero M., Acevedo J.F., Flores-Herrera O., Díaz N.F., Molina-Hernández A., García-López G., Flores-Herrera H. (2018). Secretion of heat shock −60, −70 kD protein, IL-1β and TNFα levels in serum of a term normal pregnancy and patients with pre eclampsia development. J. Cell Mol. Med..

[58] Sisti G., Kanninen T.T., Ramer I., Witkin S.S. (2015). Interaction between the inducible 70-kDa heat shock protein and autophagy: Effects on fertility and pregnancy. Cell Stress. Chaperones.

[59] Barut F., Barut A., Gun B.D., Kandemir N.O., Aktunc E., Harma M., Harma M.I., Ozdamar S.O. (2010). Expression of heat shock protein 70 and endothelial nitric oxide synthase in placental tis sue of preeclamptic and intrauterine growth-restricted preg nancies. Pathol. Res. Pract..

[60] Wataba K., Saito T., Takeuchi M., Nakayama M., Suehara N., Kudo R. (2004). Changed expression of heat shock proteins in various pathological findings in placentas with intrauterine fetal growth restriction. Med. Electron. Microsc..

[61] Ziegert M., Witkin S.S., Sziller I., Alexander H., Brylla E., Hartig W. (1999). Heat shock proteins and heat shock protein-antibody complexes in pla cental tissues. Infect. Dis. Obstet. Gynecol..

[62] Hnat M.D., Meadows J.W., Brockman D.E., Pitzer B., Lyall F., Myatt L. (2005). Heat shock protein-70 and 4-hydroxy-2-nonenal adducts in human placental villous tissue of normotensive, preeclamptic and intrauterine growth restricted pregnancies. Am. J. Obstet. Gynecol..

[63] Saghafi N., Pourali L., Ghavami Ghanbarabadi V., Mirzamarjani F., Mirteimouri M. (2018). Serum heat shock protein 70 in preeclampsia and normal pregnancy: A systematic review and meta-analysis. Int. J. Reprod. Biomed..

